# Diagnostic Difficulties in Woman with Crohn's Disease, Ascites, and Elevated Value of Serum CA125 Antigen

**DOI:** 10.1155/2014/981726

**Published:** 2014-11-23

**Authors:** Maria Kłopocka, Ariel Liebert, Joanna Bielińska, Marcin Manerowski

**Affiliations:** ^1^Department of Gastroenterological Nursing, Medical College, Nicolaus Copernicus University, Ujejskiego 75, 85-168 Bydgoszcz, Poland; ^2^Outpatient Department for Inflammatory Bowel Diseases, The Jan Biziel University Hospital No. 2, Ujejskiego 75, 85-168 Bydgoszcz, Poland

## Abstract

Variety of symptoms and atypical clinical course of Crohn's disease (CD) often create the need for additional diagnostic procedures. In the described case of woman with CD, there was a suspicion of coexistence of ovarian cancer. This issue is particularly important in patients treated with immunosuppressants and biological agents. The discussion focused on the usefulness of CA125 (cancer antigen 125, mucin 16) serum level estimation in clinical practice and draws attention to the possible reasons for the increase of its value which is not associated to ovarian cancer.

## 1. Introduction

Crohn's disease (CD) is a chronic, incurable inflammatory condition, which may be localized in any part of the gastrointestinal tract but mainly in terminal ileum and colon. Exact etiology and pathogenesis are still not clear, which makes this disease an important diagnostic and therapeutic problem [1, 2]. Correct diagnosis of this condition usually requires the cooperation of specialists from various fields of medicine, as well as different diagnostic procedures such as endoscopy with microscopic examination of tissue samples, imaging techniques, and laboratory tests. Variety of symptoms and atypical clinical course of the CD often create the need for additional diagnostic procedures [2, 3]. CA125 (cancer antigen 125, mucin 16), the protein classified as a family of mucin member, has been intensively studied because of its wide use in clinical practice as tumor marker. The increased serum concentration of this antigen occurs in over 80% of women with ovarian epithelial cancer. Changes in the serum concentration of mucin 16 are considered as a reliable indicator of response to treatment as well as progression of the disease. There is still lack of agreement among researchers on the molecular and biochemical nature of CA125 [4, 5]. This macromolecular glycoprotein, whose function is still not fully understood, is encoded by a gene MUC16 located on chromosome 19. Several studies indicate a relationship between CA125 and the immune system. It is speculated that CA125 can act as a barrier to protect epithelial cells against various infectious agents and physical injury [6–8]. Increasingly, attention is also drawn to the possibility of participation of mucin 16 in some processes resulting in progression of the disease. According to some researchers, this molecule may facilitate adhesion of cancerous cells to healthy cells, thereby accelerating the occurrence of metastasis. It has been shown that CA125 has the ability to inhibit NK (natural killers) cell response, which may result in tumor cell protection against the immune response of the organism. It is also possible that antigen positively affects the growth and motility of malignant cells and may increase their resistance to applied treatment [6, 9, 10].

## 2. Case Report

32-year-old woman with CD localized in the small intestine and in the ileocaecal valve has been treated in the conventional manner (azathioprine (2,5 mg/kg), mesalamine (2 g a day) and budesonide (9 mg a day)) for half a year. The diagnosis of the disease was confirmed by colonoscopy, abdominal ultrasound examination, and microscopic evaluation of samples taken during colonoscopy. In the past, the patient was treated by a gynecologist because of chronic ovaritis. She was not suffering from any other diseases and the results of laboratory tests showed only moderate anemia and elevated CRP (C-reactive protein) level.

Due to the ineffectiveness of such therapy (abdominal pain, diarrhea), she received biological treatment, anti-TNF*α* (tumour necrosis factor *α*), infliximab (at a dose of 5 mg/kg) intravenously at weeks 0, 2, and 6 with the outcome of clinical and endoscopic remission. Treatment with azathioprine (2,5 mg/kg) and mesalamine (2 g a day) was continued during the remission period.

**Figure 1:**
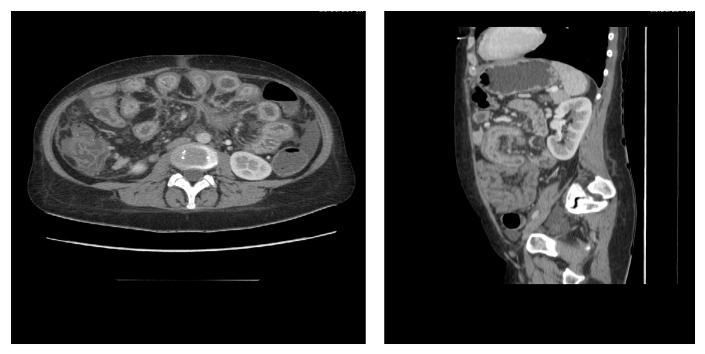
Abdominal computed tomography (CT). CT of the abdomen shows ascites. Liver, pancreas, spleen, and kidneys look normal. Thickened wall of sigmoid and ascending colon, cecum, and a significant part of the ileum, inflammatory changes. Numerous, enlarged mesenteric lymph nodes.

After three years, the disease course worsened. In addition to the severity of diarrhea and abdominal pain, an increasing abdominal circumference has been observed, as well as swelling of lower extremities. The patient returned to the gynecologist for consultation. Due to suspicion of ovarian cancer with concomitant ascites, numerous laboratory tests were performed, which showed a decrease of total protein, including albumin fraction, and an increase of CRP and CA125 level, which exceeded the normal upper limits more than five times (Table 1). Abdominal ultrasonography and CT (computed tomography) showed the presence of ascites, as well as wall thickening with features of inflammation in the jejunum (Figure 1). Apart from the so far used mesalamine and azathioprine, budesonide was included to the treatment at a dose of 9 mg a day. Additional tests were performed to rule out infection, tuberculosis, kidneys and liver diseases. Within two months there was no improvement in general condition of the patient. Ascites was still present, as well as abnormal serum levels of albumin, CRP, and CA125. Due to persistence suspicion of malignancy, a diagnostic laparoscopy was ordered by gynecologist and oncologist, preceded by a transvaginal ultrasound scan, to clearly exclude ovarian cancer coexistence. During laparoscopy, the fluid was collected from the abdomen to the laboratory and microbiological evaluation. The test results indicated a transudate, with no signs of infection. A negative result of diagnostic laparoscopy influenced the decision to intensify Crohn's disease treatment. The patient received anti-TNF*α*, adalimumab (at a dose of 160, 80, and then 40 mg every other week). The choice of the drug was dictated by patient preference, because of everyday business activity. After a period of 14 weeks, clinical remission was achieved, as well as recovery of ascites and normalization of CA125 serum level (Table 1). Maintenance treatment with adalimumab was continued for one year. The clinical remission has been maintained for 18 months so far since the completion of biological treatment.

## 3. Discussion

Due to the low specificity, antigen CA125 estimation cannot be regarded as routine disease marker in women suffering from ovarian cancer. There are numerous reports of increased serum levels of mucin 16 in the course of proliferative diseases of many organs, not necessarily related to gynecological oncology. Depending on the analyzed clinical entity, there are few reports published as case reports or observations carried out on a large group of patients [7, 8, 11, 12]. The usefulness of CA125 as a biomarker for lung cancer has been reported, as well as its strong predictive value in the diagnosis of peritoneal metastasis in patients with gastric cancer [13–15]. Kouba et al. suggested the clinical usefulness of serum CA-125 levels measurement as a predictor of response to treatment in patients undergoing radical cystectomy for transitional cell carcinoma of the bladder [16]. Increased concentration of mucin 16 was also observed in the course of lymphoma, melanoma, malignant diseases of pancreas, liver, breast, colon, and rectum [7, 12]. Increase in the serum concentration of the analyzed agent was also found in numerous diseases having no neoplastic etiology, among them, in the course of renal failure, sarcoidosis, acute pancreatitis, pelvic inflammation, various pleura, and peritoneum infectious diseases and decompensated liver diseases [6–8, 12, 17]. In recent years the potential usefulness of the CA125 level measurement in cardiac diseases was reported [8, 18]. According to the authors, the serum antigen level can be used as an independent predictor of death in patients hospitalized with acute heart failure [19, 20]. Probably CA125 value can also be used as a prognostic marker of long-term hospitalization and occurrence of cardiovascular events resulting from varying degrees of heart failure [21].

The reasons for the increase of CA125 concentration in so many different diseases are still not fully understood. According to current knowledge, this mucin may be produced continuously in small amounts by different cells [12]. The existing malignancy may be a factor contributing to the increase of its production. It was also suggested that mesothelial cells can be a source of antigen, even without the accumulation of fluid in the pleural, pericardial, and peritoneal cavities [6, 8, 12]. Increased concentration of mucin 16 in the course of inflammatory diseases may result from the stimulation of the body's immune system and activation of cytokines and other inflammatory factors [19]. Probably, the increased CA125 serum level may occur in the course of every disease entity, complicated by the occurrence of ascites [8, 12, 22, 23]. In the described case of a woman with CD, exacerbated inflammatory process affected significant segments of the small intestine, leading to diarrhea, malabsorption, and excessive protein loss. Severe hypoalbuminemia was in turn the cause of fluid accumulation in the peritoneal cavity. Accumulation of fluid in peritoneal cavity increases pressure exerted on mesothelial cells which may result in higher CA125 secretion and its elevated serum level. However, the occurrence of ascites in women who required gynecological treatment in the past and who was also treated with immunomodulators and infliximab due to Crohn's disease was the reason for “oncologic anxiety,” despite the absence of changes in the ovaries imaging studies (pelvic ultrasonography, CT). There is concern that these therapies may be associated with an increased risk of malignancy [24]. Intensification of treatment, including the reintroduction of anti-TNF*α*, has allowed inducing and maintaining disease remission, improved the results of laboratory tests, and led to resolution of ascites. However, such decision in this case was preceded by gynecological and oncological consultations as well as invasive procedure, diagnostic laparoscopic examination.

## 4. Conclusion

Severe small intestine inflammation in active Crohn's disease can lead to hypoalbuminemia and ascites. Nonspecific increase of CA125 (known as ovarian cancer marker in females) serum level may be a consequence of ascites, as well as gut inflammation. Such a course of disease with increasing levels of serum CA125 creates a major diagnostic problem in women with Crohn's disease.

## Figures and Tables

**Table 1:** Results of laboratory tests during exacerbation of the disease, follow-up, and clinical remission.

Parameter	Disease	Month 1	Month 2	Remission	Normal values
	exacerbation			Month 6	
HCT	37.9	38.7	39.9	42.4	% (37–47)
HGB	12.2	12.3	13.0	13.7	g/dL (12–16)
RBC	4.41	4.46	4.62	4.72	Million cells/mcL (4-5)
WBC	3.87	4.02	4.45	7.22	Cells/mcL (3,900–10,200)
PLT	366	345	331	354	Cells/mcL (130,000–400,000)
Albumin	2.18	2.48	2.57	3.35	g/dL (3.5–5.2)
CRP	18.77	12.24	10.35	5.49	mg/L (0-1)
CA 125	190.3	121.2	120.5	35.19	U/mL (<35)

HCT: hematocrit; HGB: hemoglobin; RBC: red blood cells; WBC: white blood cells; PLT: platelets; CRP-C: reactive protein; CA125: cancer antigen 125.

## Abbreviations

CD: Crohn’s disease
CA125: Cancer antigen 125
CRP: C-reactive protein
TNF*α*: Tumour necrosis factor *α*
CT: Computed tomography.
